# Soziologie – Corona – Kritik

**DOI:** 10.1007/s11609-020-00417-3

**Published:** 2020-11-18

**Authors:** Stephan Lessenich

**Affiliations:** Institut für Soziologie, Ludwig-Maximilians-Universität München, Konradstr. 6, 80801 München, Deutschland

**Keywords:** SARS-CoV‑2, COVID-19, Krise, Kapitalismus, Corona-Politik, Krise des Krisenmanagements, Soziologie, Solidarität, SARS-CoV‑2, COVID-19, Crisis, Capitalism, Corona politics, Crisis of crisis management, Sociology, Solidarity, SARS-CoV‑2, COVID-19, Crise, Capitalisme, Politique de lutte contre le coronavirus, Crise de la gestion de crise, Sociologie, Solidarité

## Abstract

Die COVID-19-Pandemie läutet kein neues gesellschaftliches Zeitalter ein. Die bislang erkennbaren Mechanismen ihrer Bearbeitung sind vielmehr Ausweis jener strukturellen sozioökonomischen und soziopolitischen Krisendynamik, die als Signatur des demokratischen Kapitalismus gelten muss. Ebenso wenig sollte das herrschende Krisenmanagement als eine die kapitalistische Akkumulations- und Profitlogik zumindest vorübergehend stillstellende „Politik des Lebens“ missverstanden werden: Es sind vielmehr nur bestimmte Leben, deren Schutz und Rettung sich die Regierungen der demokratisch-kapitalistischen Industrienationen verschrieben haben. Gegenüber einer zeitdiagnostischen Adaption der beliebten Lebensberatungssemantik der „Krise als Chance“ ist daher Zurückhaltung geboten. Eine Chance aber könnten die gegenwärtigen Umstände tatsächlich für die Soziologie bieten: Wenn sie nämlich endlich aufhören würde, ihre unvermeidlich in gesellschaftspolitische Gestaltungsprozesse involvierte Praxis als eine „unideologische“, „wertfreie“ und politisch „neutrale“ zu verkennen.

## Abstract (en)

The COVID-19 pandemic does not herald a new social era. Rather, the mechanisms of dealing with the pandemic, as far as they can be identified at this point, bear testimony to the structural socio-economic and socio-political crisis that must be regarded as the signature of democratic capitalism. Nor should the prevailing crisis management be misunderstood as a “politics of life” which (at least temporarily) suspends the capitalist logic of accumulation and of profit: as it is only certain lives that the governments of the democratic-capitalist industrialized countries are committed to saving and protecting. This means that any adoption of the life-coaching semantics of “crisis as an opportunity” should be treated with caution. However, for sociology itself the current circumstances could indeed offer an opportunity: that is, if it would finally stop denying that its own practice is inextricably enmeshed in (trans-)formative social processes and is never unideological, nor value-free, nor politically neutral.

## Résumé

La pandémie de COVID-19 n’inaugure pas une nouvelle ère sociale. Les mécanismes mis en œuvre jusqu’à présent pour y faire face sont bien plus l’expression de la dynamique de crise structurelle socioéconomique et sociopolitique qu’il convient de considérer comme la signature du capitalisme démocratique. Il ne faudrait pas non plus prendre la gestion de la crise en vigueur pour une « politique de la vie » suspendant au moins temporairement la logique capitaliste de l’accumulation et du profit : C’est bien plutôt à la protection et au sauvetage de certaines vies que les gouvernements des nations industrielles capitalistes démocratiques vouent leurs efforts. Il y a tout lieu d’être réservé quant à l’adaptation au constat sociologique de la sémantique populaire du développement personnel présentant la « crise comme une opportunité ». Cependant, les circonstances actuelles pourraient effectivement représenter une opportunité pour la sociologie : Si elle cessait de se méprendre sur sa pratique prétendument « non idéologique », « libre de jugements de valeur » et « neutre » politiquement alors que celle-ci est inévitablement impliquée dans les processus politiques de transformation sociale.

## Die COVID-19-Pandemie als Mutter aller Krisen?

Krisen sind das Lebenselixier nicht nur der kapitalistischen Produktionsweise und Gesellschaftsformation, sondern auch der kritischen Soziologie. So sehr gesellschaftliche Entwicklung unter kapitalistischen Bedingungen als Widerspruchsdynamik sich vollzieht, so sehr ist eine Gesellschaftswissenschaft mit emanzipatorischem Anspruch auf die Beobachtung eben dieser Widerspruchsbewegung fixiert. Mit einer Mischung aus Faszination und Grauen registriert sie den jeweils jüngsten Gestaltwandel des Krisengeschehens: einerseits dieses, im Interesse der von ihm am stärksten betroffenen Sozialpositionen und Weltregionen, bedauernd – und andererseits aber, im Interesse einer endlichen Überwindung des krisengenerierenden Strukturmechanismus, zumindest insgeheim auf die Zuspitzung desselben hoffend. Und so werden denn von dieser doppelt interessierten Seite immer neue Krisen identifiziert und analysiert, seien es nun solche konjunktureller oder aber (und insbesondere) struktureller Art.

Im Zuge und Zeichen der letzten „klassischen“ Krise, jener des finanzmarktdominierten Kapitalismus, die sich vor nunmehr gut einem Jahrzehnt vor allem in den kapitalistischen Zentren Europas und Nordamerikas Bahn brach, etablierte sich im deutschsprachigen Diskurs sogar die Figur der „multiplen“ oder „VielfachKrise“ (Bader et al. 2011), mit der ein über die ökonomische Akkumulationskrise hinausgehendes und -weisendes, komplexes Krisensyndrom von sozial-ökologischer Krise, Krise der Reproduktion und Krise der parlamentarischen Demokratie bezeichnet wurde. Schon damals diagnostizierte man „eine Zuspitzung von Widersprüchen der globalen Entwicklung des neoliberalen Kapitalismus“ (ebd., S. 13), deren Weiterung zu einer „Struktur- und Hegemoniekrise der derzeitigen Phase des Kapitalismus“ oder gar zu einer veritablen „Systemkrise im Sinne einer umfassenden Krise der kapitalistischen Gesellschaftsformation“ (ebd., S. 14) zwar prinzipiell offen bleiben musste, aber subtextuell eben doch als wie auch immer unwahrscheinliche historische Option ins prognostische Spiel gebracht wurde.

Und jetzt, nach dem zwischenzeitlichen Einbruch einer so nicht vorherzusehenden Migrations- sowie einer nicht länger zu verhehlenden Klimakrise in die Öffentlichkeit auch der westlichen Wohlstandsgesellschaften, also auch das noch: Jetzt auch noch Corona. Steht jetzt die definitive Kriseneskalation ins Haus? Markiert die Pandemie eine „historische Zäsur“ (Dörre 2020a, Abschn. 4), die „Möglichkeiten für einen gesellschaftlichen Pfad- und Systemwechsel“ (Rosa 2020, Abschn. 1) eröffnet? Sollte es am Ende – geradezu sciencefictioneske Ironie der Geschichte! – ein mikroskopisch kleines Virus von maximal 160 Nanometer Größe sein, das dem globalisierten, ubiquitären, in seinen sozial wie ökologisch destruktiven Effekten mittlerweile außer Rand und Band geratenen Kapitalismus den entscheidenden Schlag versetzt?

Nun: „Only time will tell“ – nichts anderes wird eine Soziologie, die bei Sinnen ist, zu dieser Suggestivfrage sagen können. Und doch, so die hier zu vertretende These, spricht aus gesellschaftsanalytischer Perspektive wenig dafür, dass mit der Entstehung und Verbreitung von COVID-19 die Mutter aller Krisen eingeläutet wäre. Krisen sind die „normale Anomalie“ (Steg 2020) der gesellschaftlichen Moderne, und die nunmehr so genannte „Coronakrise“ ist eher eine der mittlerweile vielen Töchter jener strukturellen sozioökonomischen und soziopolitischen Krisendynamik, die als Signatur des demokratischen Kapitalismus gelten muss. Wie bereits angedeutet, ist allein das vergangene Jahrzehnt mit Finanz‑, Migrations‑, Klima- und nun Pandemie-Krise Zeuge von mindestens vier globalen makrosozialen Krisen geworden, und es ist davon auszugehen, dass die nächste bereits gesellschaftssystemisch ausgebrütet wird. Insofern ist es eher unwahrscheinlich, dass auf die epochale „v. Chr.“/„n. Chr.“-Zäsur in der zukünftigen Geschichtsschreibung die pandemiebedingte Unterscheidung der späten Menschheitsgeschichte in die Zeit „vor“ und „nach Corona“ folgen wird. Nicht nur weil das Virus erst seit wenigen Monaten sein Unwesen treibt, ist Zurückhaltung in Sachen Epochenbruchdiagnostik geboten und sollte der um sich greifende „Rausch des Epochalen“ (Vogl 2020) die Soziologie misstrauisch stimmen. Sondern auch und vor allem, weil Epochenbrüche definitionsgemäß selten sind – und die jeweiligen Zeitgenoss*innen naheliegenderweise nicht die geeignetsten Urteilsinstanzen für die Geschichtsrelevanz der ihnen jeweils begegnenden Zeitläufte. „Es wird sich wahrscheinlich etwas geändert haben“ (ebd.): Allenfalls diesem zurückhaltend-zukunftsoffenen Futur II des Literaturwissenschaftlers Joseph Vogl lässt sich wohl auch soziologisch ohne Weiteres zustimmen.

Was allerdings das auch unter Soziolog*innen offenbar verbreitete Gefühl, einer Zeitenwende beizuwohnen, erklären könnte, ist die Tatsache, dass keine der zuvor genannten, jüngeren Krisen vergleichbar tief in die Lebenswelt und Alltagspraktiken der Leute eingegriffen hat. Will heißen: in das gesellschaftliche Leben der „reichen Demokratien“ (Wilensky 2002). Anders als Finanz‑, Migrations- oder Klimakrise war die jüngste Krisenerfahrung für breite Bevölkerungsmehrheiten in den westlichen Wohlstandsgesellschaften keine bloß abstrakte, nicht allein eine der anderen, sondern eine, an der auch hierzulande eigentlich niemand vorbeikam. Anders als in den Krisen zuvor hat die Coronakrise die Normalität der Lebensführung nicht nur der in kritisch-soziologischen Debatten überstrapazierten Sozialfigur „privilegierter Professoren“ (Dörre 2020b) „im Elfenbeinturm gesicherter Beamtenverhältnisse“ (Rosa 2020, Abschn. 5.2) erschüttert, sondern – gesellschaftlich ungleich relevanter – die Lebenswelt der europäischen Mittelklassen. Keine Flugreisen und keine Konzertbesuche, keine samstägliche Shoppingtour durch die Fußgängerzone und keine werktägliche Afterwork-Zusammenkunft mit den Bürokolleg*innen: Was für vier Fünftel der Weltbevölkerung – für Minenarbeiter in Brasilien und Textilarbeiterinnen in Bangladesch, für die Müllhaldenkinder in Accra ebenso wie für die migrantischen Fleischzerleger in Rheda-Wiedenbrück – immer schon selbstverständliche Entbehrungen waren, wurde mit Corona, zumindest für einen kurzen historischen Augenblick, auch für das obere Fünftel der Weltsozialstruktur (vgl. Korzeniewicz und Moran 2009) real.

Die von der Pandemie bzw. ihrer Bekämpfung über die westlichen Industriegesellschaften gebrachte „Zwangsentschleunigung“ (Rosa 2020, Abschn. 2) begegnete deren Bürger*innen als ein „hartes […] soziales und materiales Faktum“ (ebd.). Die soziologische Behauptung aber, dass dies auch ein „global beobachtbares“ (ebd.) Phänomen gewesen sei, bedarf durchaus der Qualifizierung. Denn in gewisser Weise sind weite Teile der Weltbevölkerung schon vor und ganz ohne Corona „zwangsentschleunigt“ gewesen: Den Mitgliedern der mobilen Klassen, und das sind in den reichen Gesellschaften eben auch weite Teile der „globalisierungsskeptischen Somewheres“ (Dörre 2020a, Abschn. 3), ist es beispielsweise nicht vorstellbar, dass mindestens 80 % der Weltbevölkerung noch nie geflogen sind (Kretzschmar und Schmelzer 2019) – und dies womöglich auch ein Leben lang nicht tun werden. Die jähe Entschleunigung der westlichen Mobilitätsgesellschaften selbst wiederum war nur von kurzer Dauer: Der im Wohnviertel des Autors im Juli 2020 gesichtete Werbeslogan eines Reisebüros – „Raus aus der Krise!!“ – verweist äußerst symbolträchtig darauf, dass die Bürger*innen reicher Demokratien eben eine, wie auch immer subalterne, Exit-Option haben, die der übergroßen Mehrheit der Weltbevölkerung schlichtweg nicht zur Verfügung steht.

Nur aus einer sozialen Position heraus, die *in diesem Sinne* privilegiert ist, in der nämlich Mobilität der zur Selbstverständlichkeit gewordene Normalfall und Immobilisierung die krisenhaft-existenzielle Ausnahmeerfahrung ist, kann man wohl auf die zeitdiagnostische Idee kommen, dass „wir uns angesichts der Stillstellung und Krise zentraler gesellschaftlicher Institutionen an einem […] historischen Bifurkationspunkt befinden“ (Rosa 2020, Abschn. 4): Das formelle wie informelle Agrar‑, Industrie- und Dienstleistungsproletariat dieser Welt hat schon vor langer, langer Zeit eine gesellschaftsgeschichtliche Abzweigung genommen, die einer Soziologie im europäisch-westlich-nördlichen Selbstbezug allerdings nicht nur nicht der Rede wert ist, sondern für die ihr letztlich das theoretische Rüstzeug, das empirische Sensorium und das begriffliche Instrumentarium fehlen. Doch dazu mehr und Prinzipielleres zum Ende dieses Beitrags.

Einleitend gilt es nur noch Zweifel anzubringen an der Deutung des SARS-CoV-2-Virus als „externer Schock“, als ein „Naturereignis“, das – einem aus den unendlichen Weiten des Universums auf die Erdoberfläche stürzenden Meteoriten gleich – in seiner Entstehung den gesellschaftlichen Verhältnissen äußerlich sei, d. h. „ursprünglich außerhalb gesellschaftlicher Funktionsmechanismen“ (Dörre 2020a, Abschn. 4) stehe. Dem ist ganz gewiss nicht so. Schon soziologisch nicht: Es gibt in der Weltgesellschaft, im globalisierten Kapitalismus, schlicht kein „Außen“ mehr, sondern nur noch globale Binnenverhältnisse (Kreckel 2004, S. 39 ff.). Aber auch Naturwissenschaftler*innen verweisen darauf, dass die COVID-19-Pandemie als soziales Phänomen – und *nur* als solches – zu verstehen ist: „recent pandemics are a direct consequence of human activity […]. Rampant deforestation, uncontrolled expansion of agriculture, intensive farming, mining and infrastructure development, as well as the exploitation of wild species have created a ‚perfect storm‘ for the spillover of diseases from wildlife to people“ (Settele et al. 2020). Es sind die kapitalistische Produktionsweise, der unstillbare Ressourcenbedarf der Industriegesellschaften, die „imperiale Lebensweise“ (Brand und Wissen 2017) der reichen Länder des globalen Nordens, die an der Wurzel des aktuellen Pandemiegeschehens liegen. Es sind nicht „die Natur“ oder „der Planet“, die nun in Gestalt eines heimtückischen Virus „zurückschlagen“ (Hartmann 2020). Es ist vielmehr die politisch-ökonomische Verfasstheit der Weltgesellschaft, die nicht erst jetzt, aber jetzt in nochmals verschärfter Weise sich selbst zum Verhängnis wird – und dabei insbesondere die am schwächsten Gestellten am stärksten trifft.

## Das Corona-Krisenmanagement: Geld oder Leben?

SARS-CoV-2-Virus und COVID-19-Pandemie läuten somit kein neues Zeitalter ein. Aber in der Tat machen sie „[w]ie unter einem Brennglas all jene Unsicherheiten und Ungleichheiten sichtbar, die in modernen kapitalistischen Gesellschaften seit langem (re)produziert werden“ (Dörre 2020a, Abschn. 6). Mehr noch: Sie werfen ein Schlaglicht auf das Zeitalter, in dem wir bereits leben – und beleuchten dabei nicht nur die gesellschaftlichen Verhältnisse im globalisierten Kapitalismus, sondern die gesellschaftlichen Naturverhältnisse im „Kapitalozän“ (Altvater 2018; Moore 2016). Denn es ist nicht, wie in der mittlerweile gängigen Rede vom „Anthropozän“ suggeriert, „der Mensch“, welcher Erdgeschichte schreibt, sondern der Kapitalismus bzw. die kapitalistische Produktionsweise als „die bestimmte gesellschaftliche Form, […] die ihm die Macht dazu verleiht“ (Altvater 2018). Es sind die spezifisch kapitalistische Form der ökonomischen Akkumulation, die darauf bezogenen politischen Regulationsweisen und die damit verbundenen kulturellen Normative und sozialen Praktiken, die ein Mensch-Natur-Verhältnis hervorgebracht haben, in dessen Rahmen „pandemics are likely to happen more frequently, spread more rapidly, have greater economic impact and kill more people“ (Settele et al. 2020).

In der kapitalistisch geprägten Gesellschafts- und Erdformation sind ökonomische, ökologische und eben auch epidemiologische Krisen formationscharakteristische Phänomene. Wenn also die Krise in jeweils neuer Gestalt wiederkehrt und damit als solche gewissermaßen auf Dauer gestellt ist, dann hat sich der gesellschaftsanalytische Fokus begründetermaßen nicht nur auf diese Krisendynamik selbst zu richten, sondern auf das Krisenmanagement, mit dem sie politisch bearbeitet wird – und damit wiederum auf eine Krisendynamik zweiter Ordnung, nämlich die „Krisen des Krisenmanagements“. Exakt diesen Schritt hat aber bereits vor einem halben Jahrhundert – unter Verwendung auch genau dieser Formel (Offe 1973) – die damals so genannte Spätkapitalismustheorie vollzogen. In der Quintessenz ging es ihr darum, auf die inhärenten Grenzen der Steuerungskapazitäten demokratisch-kapitalistischen Staatshandelns hinzuweisen, auf „die Defizienz und Begrenztheit stabilisierender Staatstätigkeit“ (ebd., S. 198), die sich, wie hier nur ansatzweise wird gezeigt werden können, auch und gerade in der Corona-Krise offenbaren. Zugleich war es dieser Theorie aber ein Anliegen, gegen orthodox-marxistische Zusammenbruchsfantasien darauf hinzuweisen, dass der demokratisch-kapitalistische Interventionsstaat ungeachtet seiner strukturellen Begrenzungen durchaus die – allerdings widersprüchliche und konflikthafte und daher eben *permanent prekäre* – Bestandsfähigkeit des „Systems“ zu sichern in der Lage sei.

Der analytische Clou der Theorie bestand nun darin, den demokratisch-kapitalistischen Staat *als eben solchen* ernst zu nehmen: ihn also in seinem politisch-administrativen Handeln als nicht nur durch die kapitalistische, sondern eben auch durch die demokratische Logik (sowie zusätzlich durch die staatliche Eigenlogik) bestimmt zu sehen (Borchert und Lessenich 2016). Aus dieser Perspektive ist der Staat in der Tat nicht nur „das Instrument ‚der Herrschenden‘ bzw. der herrschenden Klasse“ (Rosa 2020, Abschnitt 3), er ist nicht allein auf das Interesse des Kapitals festgelegt (so dieses überhaupt homogen und eindeutig zu identifizieren wäre). Vielmehr ist er durch Organisationsstrukturen geprägt, „deren spezifische Selektivität darauf angelegt ist, den ‚privaten‘ Steuerungsmodus der kapitalistischen Ökonomie mit den von ihr ausgelösten Vergesellschaftungsprozessen zu vereinbaren und *koexistenzfähig* zu machen“ (Offe 1973, S. 212, Hervorh. i. Orig.). Der demokratisch-kapitalistische Staat operiert damit stets in einer genuinen Doppelrolle, seine Interventionen folgen durchgängig einer doppelten Funktionalität, nämlich der institutionellen *Vermittlung* zwischen ökonomischen Produktions- und sozialen Reproduktionsinteressen bzw., wenn man so will, zwischen „Systemrelevanz und Lebensrelevanz“ (Rosa 2020, Abschn. 3). Exakt diese Vermittlungslogik aber, diese *Doppelbindung* demokratisch-kapitalistischer Staatsintervention, so behaupte ich, materialisiert sich auf handgreifliche Weise in Zeiten der Pandemie.

Was nämlich macht das gegenwärtige Krisenmanagement in den reichen Gesellschaften – namentlich in der Bundesrepublik Deutschland – aus? Auch in der politischen Linken und der kritischen Soziologie ist die Ansicht verbreitet, dass sich im Zeichen von COVID-19 der unbedingte Schutz des Lebens gegen die kapitalistische Profitlogik durchgesetzt oder jedenfalls „zeitweilig den Vorrang vor Wirtschaftlichkeitserwägungen erhalten“ (Dörre 2020a, Abschn. 5) habe. Tatsächlich aber war es nicht etwa pauschal und allgemein *das* Leben, das „für viele demokratische Regierungen“ (ebd.) plötzlich zählte. Vielmehr sind es *bestimmte* Leben, deren Schutz und Rettung sich die Exekutivgewalten der demokratisch-kapitalistischen Industrienationen verschrieben haben. Sie betreiben keine „Politik des Lebens“ ohne Wenn und Aber, sondern eine *Politik mit dem Leben*, die sich just in dem zuvor theoretisch eingeführten Spannungsverhältnis von kapitalistischer und demokratischer Logik bewegt.

Auch auf die Gefahr hin, mich dem (letztlich systemkonformen) Zynismus-Vorwurf der von der Lebensbejahung der Herrschenden positiv Überraschten auszusetzen: Was das Corona-Krisenmanagement auszeichnet, ist eine soziale Selektionslogik, die mit Foucault’schen Kategorien der Biopolitik gefasst werden könnte – letztlich entscheiden die politisch verantwortlichen Akteure, überall und allenthalben, im Sinne der Unterscheidung, „was leben soll und was sterben kann“ (Sarasin 2020; Lessenich 2020c). Die erste Selektionslinie verläuft dabei entlang der Staatsbürger*innenschaft: In der Pandemie erweist sich die vermeintlich universalistische Inklusionsinstanz der „citizenship“ wieder einmal als das, was sie eben auch und im Kern sogar vor allem ist, nämlich ein Instrument der sozialen Exklusion.

Nehmen wir das deutsche Corona-Management als Beispiel für den doppelten Standard des staatlichen Gesundheits- und Lebensschutzes: Da organisierte die Bundesregierung zu Beginn der Krise eine spontane Rückholaktion, um rund 200.000 deutsche Tourist*innen so schnell wie möglich aus ihren Urlaubsdestinationen rund um die Welt zurück in den sicheren Schoß des heimischen Gesundheitswesens zu geleiten – während es wochenlanger bürokratischer Verrenkungen bedurfte, ehe die Bundesrepublik stolz die Aufnahme von 47 unbegleiteten Minderjährigen aus der Hygienehölle der griechischen Flüchtlingslager bekanntgeben konnte (wobei sich später herausstellte, dass sie fast alle ohnehin einen rechtlichen Anspruch auf Familienzusammenführung gehabt hätten). Das Außenministerium sprach reihenweise Reisewarnungen aus – für Länder wie Pakistan, deren Besuch den Bundesbürger*innen gesundheitlich nicht zuzumuten sei, in die freilich zeitgleich bedenkenlos Sammelabschiebungen durchgeführt wurden (von Hardenberg 2020). Nach der Aufhebung der vorübergehenden Grenzschließungen und der Lockerung der Reisebeschränkungen wurde von der deutschen Öffentlichkeit das nationale Bürger*innenrecht auf touristische Mobilität – „Die Deutschen wollen die Welt erobern“^1^ – bekräftigt und zugleich darauf gepocht, dass „[w]ichtigster Grundsatz […] der Schutz der Urlauber“, nicht etwa der einheimischen Bevölkerung in den Tourismusregionen, zu sein habe: „Kliniken müssen genügend Kapazitäten vorhalten und im Notfall auch infizierte Touristen versorgen können“ (Temsch 2020). Auch in Deutschland selbst wird systematisch mit zweierlei Maß gemessen, die Hygienebedingungen etwa in Geflüchtetenunterkünften sind nicht ansatzweise vom selben administrativen Interesse wie die in Universitätshörsälen, die vorsichtshalber auch im Wintersemester geschlossen bleiben. Und bei der erst mit Corona vollzogenen politischen Skandalisierung der Arbeitsbedingungen in der deutschen Fleischindustrie stand nicht – sonst wäre dies schon früher geschehen – die Gefährdung von Leib und Leben osteuropäischer Vertragsarbeiter im Vordergrund des öffentlichen Interesses, sondern vielmehr der Schutz der Einheimischen vor einer Verbreitung des Virus.

Derartige Doppelstandards mit dem gängigen Verdikt der „moralischen Bankrotterklärung“ (Dörre 2020a, Abschn. 5.2) zu belegen, ist zwar irgendwie richtig, trifft aber doch nicht den Kern der Sache. Denn es entspricht schlicht der demokratischen Logik nationaler Staatsgesellschaften, die Lebens- und Überlebensinteressen der eigenen Staatsbürger*innen – in diesem Fall die jedes und jeder Deutschen – im Zweifel über die vitalen Interessen migrantischer Arbeiter*innen, Geflüchteter oder welches auch immer der vom deutschen (immerhin mittlerweile nur noch touristischen) Eroberungswillen betroffenen Gastländer zu stellen. Diese Hierarchisierung von „Lebensrelevanz“ entlang von Staatszugehörigkeiten ist Teil des herrschenden demokratischen Konsenses, auf den sich staatliche Intervention immer *auch* beruft und berufen kann, ja berufen muss (Torres 2020). Im deutschen Fall wurde der Vorrang des Schutzes nationalen Lebens in der COVID-19-Pandemie gleichsam von moralpolitisch höchster Stelle, dem Deutschen Ethikrat, abgesegnet, dessen Erwägungen zu „Solidarität und Verantwortung in der Corona-Krise“ sich ausschließlich um „unsere Gesellschaft“ (Deutscher Ethikrat 2020, S. 2), die vor ungeahnten Herausforderungen stehe, drehten – die Welt um „uns“ herum geriet dem unabhängigen Beratungsgremium der Bundesregierung nur insoweit in den Blick, als von dort der nunmehr herrschende „epidemiologisch begründete Imperativ“ (ebd.) herrühre.

Die Rede vom epidemiologisch Gebotenen verweist auf eine weitere – womöglich ebenfalls zynisch klingende – soziologische Deutungsmöglichkeit der Tatsache, dass der Staat in der Pandemie als oberster Lebensschützer auftritt. Und zwar auf jene funktionale Dimension, die grundsätzlich jeder staatlichen Sozialpolitik zu eigen ist: Der Staat setzt die Reproduktionsfähigkeit menschlichen Lebens letztlich *auch* im wohlverstandenen Eigeninteresse des Kapitals – und gegebenenfalls sogar gegen dessen Widerstreben – durch (Lenhardt und Offe 1977). Aus solch funktionaler Perspektive zielte schon die Entstehung eines modernen Gesundheitswesens zuallererst auf die Herstellung bzw. Wiederherstellung der Arbeitsfähigkeit (und, nicht zu vergessen, Wehrfähigkeit) jener übergroßen Bevölkerungsmehrheit, die von der kapitalistischen Verwertbarkeit ihres Arbeitsvermögens lebt. Die Tatsache, dass in einer kapitalistischen Ökonomie der Gesundheit „Vorrang vor Wirtschaftlichkeitserwägungen“ eingeräumt wird, ist also einerseits „alles andere als selbstverständlich“ (Dörre 2020a, Abschn. 5) – andererseits aber, im Rahmen entwickelter Wohlfahrtsstaatlichkeit, ein Ausdruck nicht nur der demokratischen, sondern eben auch der kapitalistischen Logik staatlicher Intervention.

Auch hier freilich gilt die Norm des Lebensschutzes alles andere als ungebrochen, wird die bereits erwähnte Selektionslogik der „citizenship“ ergänzt um jene von „class“ und „gender“, „race“ und „age“. Kein Staat dieser Welt – *keiner* – hat sich das Leben bzw. Überleben der Armen auf die pandemiepolitischen Fahnen geschrieben, *überall* sind die Hauptbetroffenen von Krankheit und Tod die Angehörigen jener Sozialmilieus, die über geringe Einkommens- und Bildungsressourcen verfügen, mit prekären Arbeits- und beengten Wohnverhältnissen konfrontiert sind (Pitzke et al. 2020). Dies gilt, bei allen Unterschieden im Einzelnen, im Grundsatz ganz genauso für „bonapartistische“ (Dörre 2020a, Abschn. 3) wie für „intakte“ Demokratien, für die Verhältnisse in den USA und Brasilien ebenso wie für die hierzulande (Stötzel et al. 2020). Überall zeigt sich auch jener Benachteiligungsnexus von „class“ und „race“, den als „rassistische Konnotation“ (Dörre 2020a, Abschn. 5.2) von coronabezogener Klassenpolitik zu umschreiben geradezu freundlich erscheint: Der Corona-Diskurs ist durchgängig rassifiziert, Donald Trumps Rede von der „Kung Flu“ und Armin Laschets Projektion der Virusproblematik auf „Rumänen und Bulgaren“ nehmen sich in dieser Hinsicht nichts. Nicht nur in New York City waren es die migrantischen, häufig weiblichen Dienstleistungsarbeiter*innen, die die Stadt am Laufen hielten, während die Einheimischen, die es sich leisten konnten, aufs Land und ans Meer flohen (Hu und Schweber 2020) – in London und Paris, Rom oder Madrid gestaltete sich die sozialstrukturelle Verteilung von Freiheit und Zwang kaum anders. Und selbst in Spanien, dessen drakonisches Corona-Regime als das diametrale Gegenmodell zum „failed corona-state“ USA gelten kann, war es mit dem vermeintlich obersten staatspolitischen Gebot, dem Schutz der Alten, am Ende nicht so weit her: In dem austeritätspolitisch angegriffenen Gesundheitswesen waren auf dem Höhepunkt der Krise Triage-Entscheidungen zu Lasten der auch in diesem Sinne vulnerabelsten Gruppen an der Tagesordnung (Peinado 2020).

Wie auch immer man es also dreht und wendet: Es ist keineswegs so, dass im Zuge des Corona-Krisenmanagements die Relevanz „des Lebens“ systematisch jene „des Systems“ übertrumpft hätte. Die drei zentralen Steuerungsressourcen des modernen Interventionsstaates – fiskalische Mittel, administrative Rationalität, Massenloyalität (Offe 1973, S. 217 ff.) – wurden allesamt und je auf ihre Weise im Sinne der demokratisch-kapitalistischen Doppellogik eingesetzt. Jede demokratisch legitimierte und damit immer auch unter politischem Legitimationsdruck agierende Regierung verschrieb sich – und sei es auch nur rhetorisch – dem Schutz ihrer Bürger*innen; und je überzeugender sie sich diesen gegenüber als Garantin gesellschaftlicher Sicherheitsinteressen darstellen konnte, umso größere krisenvermittelte Reputationsgewinne vermochte sie für sich zu verbuchen. Jede von der Funktionsfähigkeit ihrer Nationalökonomie abhängige und damit unter politischem Akkumulationsbeförderungszwang stehende Regierung sah sich aber zugleich gehalten, mit massiven Konjunkturprogrammen sowie Hilfsmaßnahmen für einzelne Unternehmen und ganze Branchen die Wirtschaftstätigkeit so effektiv wie möglich zu stabilisieren bzw. wieder anzukurbeln, sprich als Garantin ökonomischer Profitabilitätsinteressen aufzutreten. Und mit der schrittweisen, potenziell lebensgefährdenden Aufhebung der zu Beginn der Pandemie verfügten Beschränkungen des gesellschaftlichen und wirtschaftlichen Verkehrs reagierten die Exekutiven der demokratisch-kapitalistischen Welt gleichermaßen auf die Forderungen von Bürger*innen *und* Unternehmer*innen, Konsument*innen *und* Produzent*innen nach Wiederherstellung ihrer Handlungs- und Bewegungsfreiheit.

In dieser doppelten Determiniertheit des staatlichen Krisenmanagements erweist sich die Corona-Politik eindrucksvoll als Ausdruck einer neosozialen Regierungsweise, in der von den gesellschaftlichen Subjekten ein Lebensführungsmodus sozialverantwortlicher Eigenverantwortung gefragt ist (Lessenich 2008). Jede*r ist aufgefordert, sich selbst zu steuern und zu regulieren, zu zügeln und zu kontrollieren – im Sinne und Dienste des Gemeinwohls. In Zeiten von Corona heißt das: Daheimbleiben, Abstand halten, Hände waschen, Maske tragen, Kontakte minimieren – zum Schutz der Allgemeinheit vor einem sie potenziell gefährdenden Selbst. Mit dieser spezifischen Form der Subjektivierung des Sozialen geraten die gesellschaftlichen Ursachen der Krise tendenziell aus dem Blick, und zwar nicht nur, wie bereits erwähnt, im Sinne des durch die expansiv-destruktive industriekapitalistische Wirtschafts- und Lebensweise strukturell erhöhten Pandemierisikos. Entproblematisiert werden durch die Einforderung gemeinwohldienlichen Individualverhaltens auch die infrastrukturellen Defizite eines über Jahrzehnte hinweg auf Rationalisierung und Profitabilität getrimmten öffentlichen Wohlfahrtssektors. In der deutschen Debatte um die Bekämpfung des Corona-Virus etwa fungierten die viel zitierten „Belastungsgrenzen des Gesundheitssystems“ bis zuletzt als quasi-naturgegebene Größe, von der alle pandemiebezogenen Staatsinterventionen und Verhaltensmaßregeln abzuleiten waren – als ob diese Grenzen nicht selbst politisch herbeigeführt worden wären und im gesellschaftlichen Bedarfsfall nicht auch politisch verschoben werden könnten.

Das umfassende – rechtliche, administrative, polizeiliche – Kontrollregime zur Sicherstellung sozialverantwortlicher Eigenverantwortung wiederum gewinnt seine Akzeptanz und Legitimität maßgeblich durch seine moralische Aufladung mit dem bis vor kurzem noch als verstaubt geltenden Wertbegriff der „Solidarität“ (Lessenich 2020a, 2020b). Dank COVID-19 war der Begriff plötzlich in aller Munde – in dem der politisch Verantwortlichen ebenso wie jenem der intellektuellen Deutungseliten, aber nicht minder auch in dem einer pandemisch gleichermaßen mobilisierten wie demobilisierten Zivilgesellschaft. Für die Entleerung und damit effektive Entwertung eines Hochwertbegriffs durch konsequente Inflationierung und Überdehnung seiner Verwendung sind die unzähligen Spielarten einer vermeintlichen „Corona-Solidarität“ ein geradezu idealtypisches Beispiel. Mit Blick auf das Neosoziale an der Pandemiefolgenphänomenologie aber ist vor allem von Bedeutung, dass Solidarität in Zeiten von Corona zum privaten Ausfallbürgen einer öffentlichen Verantwortung für das Soziale wird – und zum mikropolitischen Schmiermittel eines gesellschaftlichen Gestaltungsregimes, das sich gerade durch seine institutionalisierte Asozialität charakterisiert (Byers 2020).

## Die Krise als Chance (wenigstens für die Soziologie)?

„Krisis“ ist bekanntlich ein Begriff aus der Medizin, wo er die plötzliche, schockartige Verschlechterung des Gesundheitszustandes eines Erkrankten beschreibt. Gesundheitliche Krisen stellen tatsächlich eine existenzielle Wegscheide dar, für den menschlichen Körper ist die Krise eine Entscheidungssituation über Wohl und Wehe, Leben oder Tod. In diesem Sinne lässt sich auch in der Corona-Pandemie davon sprechen, dass jede individuelle COVID-19-Erkrankung einer Chance gleichkommt – der Eventualität nämlich, *nicht* daran zu sterben und sogar Immunität gegen eine neuerliche Infektion zu entwickeln. Wie bereits angesprochen, ist auf dieser Ebene die soziale Chancenstruktur allerdings eine höchst ungleiche: Sowohl das Risiko einer Ansteckung wie auch die Überlebenschance, nicht zuletzt die Möglichkeit, einen milden Krankheitsverlauf alltagspraktisch zu verarbeiten, sind alles andere als zufällig – oder gar gleich – verteilt.

Weil die Corona-Krise und deren Überwindung, jenseits der hierzulande von allen Beteiligten zu duldenden Einschränkungen der Lebensqualität, rund um den Globus viel Leid und Not, ja hunderttausendfachen Tod mit sich gebracht haben und auch weiterhin bringen werden, sollte es sich eigentlich von selbst verbieten, diesbezüglich die luxurierende Lebensberatungssemantik und abgedroschene Marketingmetaphorik der „Krise als Chance“ im Mund zu führen. Was für „das Leben“ gilt, muss freilich nicht automatisch auch für „das System“ gelten: Vielleicht ist ja die Krise eine Gelegenheitsstruktur für makrosozialen Wandel, ja gar für eine intentional herbeigeführte Gesellschaftstransformation? Wer dies bezweifelt, muss nicht gleich des bösen Willens oder arroganter Hybris bezichtigt werden. Soziologisch betrachtet ist es, aufgrund der bekannten und oben auch teilweise bereits genannten strukturierten und strukturierenden Strukturen, schlichtweg reichlich unwahrscheinlich, dass die aktuelle Krise, wie schon die parallel zu ihr ja weiterhin schwelenden Vorgängerkrisen, zu etwas grundlegend anderem als zu allfälligen, und natürlich irgendwie metamorphotischen, Verlängerungen des gesellschaftlich Gewordenen, Gegebenen und Bestehenden führen wird.

Doch seien wir einfach mal, und sei es nur um der an dieser Stelle gefragten Debattendramaturgie willen, zwanghaft optimistisch: Wo könnte denn der Keim des Transformativen in der Corona-Krise angelegt sein? Paradoxerweise gilt es selbst im Lichte dieser Rahmung zunächst auf das viele Wasser hinzuweisen, das sich im Wein eines potenziellen, pandemiegetriebenen „Paradigmenwechsels“ (Rosa 2020, Abschn. 4) findet. Denn es ist ja schlechterdings nicht von der Hand zu weisen, dass das mit dem Auftreten des Virus massiv gewachsene soziale Verlangen nach Sicherheit und Führung einerseits zu einer Fixierung auf die politische Exekutive geführt hat, die das Prinzip parlamentarischer Regierungskontrolle selbst in gefestigten Demokratien nicht nur vorübergehend ausgehebelt hat und etwa in der Bundesrepublik eine jeder Transformationsaffinität unverdächtige Figur wie Markus Söder ins Kanzleramt spülen könnte.^2^ Andererseits hat die Krise dem schon zuvor, im Zeichen namentlich der Migrationskrise, verbreiteten politischen Verlangen nach Schutz und Stärkung der nationalen Sozialgemeinschaft nochmals erheblichen Auftrieb gegeben. „Der narzisstische Wunsch nach Safe Places ist nun epidemiologisch gestützte Staatstheorie“ (Rau 2020), die aus den politischen Führungsetagen beschworene wie auch zivilgesellschaftlich sich hashtagreich selbst bestätigte Solidarität hat offen inzestuösen Charakter angenommen: Hauptsache, „wir“ halten zusammen und können uns angesichts der konkurrenzlos hohen Zahl der Intensivbetten in Deutschland gegen den Angriff des heimtückischen Virus gewappnet wähnen.^3^

Und dennoch, trotz alledem: In der erkennbaren Begrenztheit und Selbstbezüglichkeit der Corona-Solidarität scheint doch zugleich die Möglichkeit einer anderen, weitergehenden gesellschaftlichen Praxis auf. Die Ahnung davon, was Solidarität auch meinen könnte – und, wenn man für einen Moment die gewohnten Fesseln des Denkens in Alternativen ablegt, wie eine andere Form der Vergesellschaftung aussehen könnte. Dann nämlich sieht man ein öffentliches Gesundheitswesen, das auf die zuverlässige und frei zugängliche Sicherung der existenziellen Bedarfe der gesamten Bevölkerung hin ausgerichtet und ausgestattet ist; eine Wirtschaftspolitik, die systematisch nicht einer Ökonomie des Profitablen und Überflüssigen, sondern des Nötigen und Lebensnotwendigen den Vorzug gibt; schließlich eine Gesellschaftspolitik, die den Bürger*innen die sozialen Bedingungen und Bedingtheiten ihrer persönlichen Freiheiten in Erinnerung ruft und die institutionellen Voraussetzungen schafft für eine in das Wissen um die Bedarfe und Bedürfnisse der globalen Mitbürger*innenschaft eingebettete Solidarität unter Ungleichen.

Zugegeben, derartige gesellschaftspolitische Blütenträume wirken in ihrer Ambition und Abstraktion aus der Zeit – auch der Corona-Zeit – gefallen. Doch lassen sich, etwa in Gestalt der griechischen Sozialklinikbewegung, gerade im Feld der Gesundheitsversorgung gewissermaßen vor der eigenen Haustüre Vorboten einer solidarisch-universalistischen Organisationsform gesellschaftlicher Grundbedürfnisse finden, die einer „Politik des Lebens“ tatsächlich nahekommen (Chatzimichali 2020). Noch viel naheliegender wäre es für die Pandemie beobachtende Soziolog*innen freilich, nach möglichen transformativen Dynamiken der Corona-Krise nicht vor, sondern *hinter *der eigenen Haustüre zu suchen, im Feld soziologischer Wissensproduktion und Wissenschaftspraxis selbst. Zwei veritable Revolutionierungen des soziologischen Selbstverständnisses stünden nach meinem Dafürhalten mit allerhöchster Dringlichkeit an – und zumindest die fachinterne Bewusstwerdung eben dieser Dringlichkeit könnte durch die COVID-19-Pandemie befördert werden.

Die eine Revolution wird der Soziologie seit nunmehr mindestens zwei Jahrzehnten auf die Fahnen geschrieben (z. B. Wimmer und Schiller 2003) – allein, ihr westlich-eurozentrischer Mainstream wehrt sich nach Kräften dagegen, den in das soziologische Denken eingeschriebenen Strukturnationalismus zu überwinden. Das SARS-CoV-2-Virus jedoch macht unmissverständlich deutlich, wie überfällig die analytische wie normative Entnationalisierung der Soziologie ist. Und dies nicht nur, weil sprichwörtlich jedes Kind weiß, dass das Virus ein genuin globales soziales Phänomen darstellt. Sondern auch und insbesondere deswegen, weil aus einer nicht-nationalen Perspektive erschreckend deutlich wird, wie und wo die Gesellschaften Europas konsequent mit doppelten Standards operieren. Und das, wo sie doch für all die Krisen, die der Welt zuletzt widerfahren sind, maßgeblich selbst verantwortlich zeichnen. Man kann die globalen Zusammenhänge kaum überzeichnen: Die demokratischen Nationen des euroatlantischen Raums sind als kapitalistische Beutegemeinschaften verfasst. Ihre wirtschaftliche Produktivität beruht auf einer sozial-ökologischen Destruktivität, die seit Jahrzehnten, wenn nicht Jahrhunderten, zuallererst der Rest der Welt zu spüren bekommt (Lessenich 2016). Die unendlich irrationale Rationalität ihrer Produktions- und Konsumweisen bringt jene Verwerfungen hervor, die die Welt in Atem halten – Finanz- und Migrations‑, Klima- und Pandemiekrisen – und bei denen am Ende jene das größte Leid davontragen, die zu diesen Verwerfungen am wenigsten beigetragen haben. Der Soziologie, von Beginn an ein Medium der Selbstbeobachtung der westlichen „Herrengesellschaften“, stünde es gut zu Gesicht, der konstitutiven Transnationalität des gesellschaftlichen Geschehens endlich auch intellektuell Herr zu werden.

Die zweite soziologische Revolution könnte durch die offenkundigen Statusinkonsistenzen einer Virologie vorangetrieben werden, die in Zeiten von Corona zur veritablen Staatswissenschaft avanciert ist, dieser objektiven Position zum Trotz aber öffentlich auf der Betonung ihrer reinen Zulieferfunktion von Wissensbeständen für deren eigenlogische Weiterverwertung im politischen Feld meint bestehen zu müssen. Genau das aber ist die gesellschaftliche Rolle, die zuletzt verstärkt auch eine Soziologie für sich reklamiert, die in Verkennung ihrer eigenen Praxis von sich behauptet, nichts anderes als positives, analytisch-empirisches Wissen für eine evidenzbasierte Politik zu produzieren. Diesen sozialwissenschaftlichen Selbstbetrug als eben solchen zu benennen, und damit im Sinne einer solchen Benennungspraxis eine disziplinäre *Wahrheitspolitik* zu betreiben, muss eines der obersten Anliegen einer kritischen Soziologie sein: Wer heute als Soziolog*in immer noch – oder historisch betrachtet eher wieder – von sich behauptet, entweder nur ideologiefreies politisches Steuerungswissen oder aber umgekehrt komplexitätssensibles Wissen über die Unmöglichkeit politischer Steuerung zu liefern, lügt sich kollektiv und individuell in die Tasche. Denn soziologische Wissensproduktion ist immer, ob sie dies nun wahrhaben will oder nicht, ein Einsatz im Spiel der Gestaltung gesellschaftlicher Verhältnisse. Die – sei es interessierte, sei es unbedarfte – Imagination einer „autonomen“ Sphäre „rein“ wissenschaftlicher Wahrheitssuche ist de facto ein politischer Akt der Entpolitisierung einer unvermeidlich politischen Praxis. Wer soziologisches Wissen produziert, *ist* nolens volens gesellschaftsgestaltend engagiert – und damit *teilnehmende* Beobachter*in des politischen Geschehens. Zwar können sich Soziolog*innen auch, in einem Akt paradoxaler, von einer am Schein wissenschaftlicher „Neutralität“ interessierten Öffentlichkeit allerdings goutierter und honorierter Selbstverleugnung gegen das eigene Engagiertsein engagieren (vgl. de Lagasnerie 2018, S. 27) – die Akademie ist, wie wir wissen, voll davon. Aber eine kritisch sich verstehende Soziologie sollte eine derartige Entpolitisierungspolitik immer als das sichtbar machen, was sie ist: als ein – jedenfalls in der professoral bestallten Soziologie nicht einmal notwendiges – falsches Bewusstsein.

Unter kritischen Soziolog*innen aber, die mit der Anerkennung des unhintergehbaren Politismus ihrer wissenschaftlichen Praxis kein erkenntnistheoretisches Problem haben, ist es nicht gerade standesgemäß (und schon gar nicht originell), sich wechselseitig der „neuen deutschen Ideologie“ (Dörre 2020a, Abschn. 6.3) bzw. der Komplizenschaft mit der Reaktion (vgl. Rosa 2020, Abschn. 5.2) zu bezichtigen. In gesellschaftshistorisch Verzweiflung nahelegenden Zeiten ist es weder verwerflich, nach Chancen eines politisch-sozialen Paradigmenwechsels zu fahnden, noch konterrevolutionär, an die Schwerkraft des herrschenden Vergesellschaftungsmodus zu erinnern. Richtig ist aber sehr wohl, dass weder ein fröhlicher Possibilismus noch ein zynischer Defätismus das im Zeichen der Corona-Pandemie soziologisch Gebotene sind – denn letzten Endes erweisen sich beide soziologischen Haltungen als Mittelsmänner der Affirmation.^4^ Was soziologiestrategisch ansteht, ist hingegen ein *kapitalistischer Realismus*, der nicht in der Überzeugung von der vermeintlichen Alternativlosigkeit der herrschenden Verhältnisse aufgeht (Fisher 2009), sondern von der nicht nur institutionellen, sondern auch psychosozialen Verankerung des real existierenden Kapitalismus in den Strukturen der sozialen Welt und in den Praktiken der gesellschaftlichen Subjekte ausgeht – und in seinem transformativen Anliegen an beiden Seiten kapitalistischer Vergesellschaftung ansetzt.

## Footnotes

1 So der Präsident des Deutschen Reiseverbandes, Norbert Fiebig, im Interview mit dem Deutschlandfunk (Fiebig und Heimlein 2020).

2 Bemerkenswert in diesem konkreten Zusammenhang ist, dass ein äußerst öffentlichkeitswirksamer Vertreter der Soziologie wie Armin Nassehi, den erst die Corona-Krise über die Eingriffsmöglichkeiten staatlicher Politik in einer vermeintlich spitzen- und zentrumslosen Gesellschaft aufklären musste (vgl. Rosa 2020, Abschn. 3), sich auf dieses Damaskuserlebnis hin einem Medienbericht zufolge sogleich der Anschlussüberlegung widmete, „wie der starke Anführer aussehen sollte, den die Menschen jetzt brauchen“ – und das entsprechende Anforderungsprofil bestmöglich in der Person des bayerischen Ministerpräsidenten realisiert sah (vgl. Deininger und Schnell 2020, S. 3).

3 Laut OECD-Statistik kommen in Deutschland auf 100.000 Einwohner*innen 33,9 Intensivbetten, in Spanien oder Italien hingegen nur 9,7 bzw. 8,3 (OECD 2020). Werte, von denen „Entwicklungsländer“ rund um den Globus, und insbesondere in Afrika, wiederum nur träumen können: In Mali etwa gibt es sechs Intensivbetten für 18 Millionen Menschen, in Ruanda acht für zwölf Millionen (Monath 2020).

4 Ich danke Konstantin Klur für diese Einsicht.
